# CsrA Coordinates Compatible Solute Synthesis in Acinetobacter baumannii and Facilitates Growth in Human Urine

**DOI:** 10.1128/Spectrum.01296-21

**Published:** 2021-11-03

**Authors:** Josephine Joy Hubloher, Kim Schabacker, Volker Müller, Beate Averhoff

**Affiliations:** a Department of Molecular Microbiology & Bioenergetics, Institute of Molecular Biosciences, Goethe-University Frankfurt am Main, Frankfurt, Germany; The National University of Singapore and the Genome Institute of Singapore

**Keywords:** *Acinetobacter baumannii*, CsrA, carbon metabolism, osmoadaptation

## Abstract

CsrA is a global regulator widespread in bacteria and known to be involved in different physiological processes, including pathogenicity. Deletion of *csrA* of Acinetobacter baumannii strain ATCC 19606 resulted in a mutant that was unable to utilize a broad range of carbon and energy sources, including amino acids. This defect in amino acid metabolism was most likely responsible for the growth inhibition of the Δ*csrA* mutant in human urine, where amino acids are the most abundant carbon source for A. baumannii. Recent studies revealed that deletion of *csrA* in the A. baumannii strains AB09-003 and ATCC 17961 resulted in an increase in hyperosmotic stress resistance. However, the molecular basis for this observation remained unknown. This study aimed to investigate the role of CsrA in compatible solute synthesis. We observed striking differences in the ability of different A. baumannii strains to cope with hyperosmotic stress. Strains AB09-003 and ATCC 17961 were strongly impaired in hyperosmotic stress resistance in comparison to strain ATCC 19606. These differences were abolished by deletion of *csrA* and are in line with the ability to synthesize compatible solutes. In the salt-sensitive strains AB09-003 and ATCC 17961, compatible solute synthesis was repressed by CsrA. This impairment is mediated via CsrA and could be overcome by deletion of *csrA* from the genome.

**IMPORTANCE** The opportunistic human pathogen Acinetobacter baumannii has become one of the leading causes of nosocomial infections around the world due to the increasing prevalence of multidrug-resistant strains and their optimal adaptation to clinical environments and the human host. Recently, it was found that CsrA, a global mRNA binding posttranscriptional regulator, plays a role in osmotic stress adaptation, virulence, and growth on amino acids of A. baumannii AB09-003 and ATCC 17961. Here, we report that this is also the case for A. baumannii ATCC 19606. However, we observed significant differences in the Δ*csrA* mutants with respect to osmostress resistance, such as the AB09-003 and 17961 mutants being enhanced in osmostress resistance whereas the ATCC 19606 mutant was not. This suggests that the role of CsrA in osmotic stress adaptation is strain specific. Furthermore, we provide clear evidence that CsrA is essential for growth in human urine and at high temperatures.

## INTRODUCTION

Species of the genus Acinetobacter are widespread in nature and found in terrestrial ecosystems, including extreme environments, as well as in animals and humans (1–8). This requires far-reaching adaptations to very different habitats to use different carbon and electron sources, to grow at different physicochemical parameters such as pH and water activity, to adhere and built biofilms on very different substrates, and to combat host defense and antibiotic pressure (2, 3, 9, 10). Monitoring these environmental changes and responding to them is a prerequisite for the enormous ecological fitness of Acinetobacter strains. While Acinetobacter species are commonly unable to utilize glucose as a carbon and energy source, they were instead found to metabolize amino acids, lipids, aromatic compounds, long-chain dicarboxylic acids, alcohols, carnitine, intermediates of the tricarboxylic acid cycle (TCC), such as succinate, and a few sugars, such as arabinose (11–16). While the metabolic routes enabling growth on aromatic compounds and natural transformability of Acinetobacter species caught the interest of researchers in the first place, these days, human-pathogenic species come into focus since they are a major concern for global health care systems (2, 10, 17, 18). Acinetobacter baumannii is the most prominent pathogenic representative of the genus, and even though A. baumannii is an opportunistic pathogen, it became a troublesome nosocomial pathogen with increasing success in hospital settings—especially for critically ill persons in intensive care units (2, 17, 19–22).

The molecular mechanisms that enable A. baumannii to infect humans and to persist in hospital settings are only partially understood (23). It seems that the ability of A. baumannii to infect the human host is multifunctional, including the ability to adhere to biotic surfaces, to build biofilms, to evade the immune system, and to react to rapid changes within the human body (23–26). That involves coping with changes in temperature, the availability of carbon sources and nutrients, and variations of the osmotic potential (24, 25, 27–31). Human urine, for example, represents a harsh environment with a high osmotic potential and limited nutrients (32, 33). However, urinary tract infection is one of the most common hospital-acquired infection, and A. baumannii has been reported to grow in human urine (34, 35). The response to hyperosmotic stress, as encountered in urine, has been studied in A. baumannii in recent years (16, 31–41). A. baumannii accumulates so-called compatible solutes (36, 39). An increase in the cytoplasmic solute content causes a decrease of the concentration gradient between the intra- and the extracellular medium; thereby, the loss of cellular water is reduced, and cell-shrinking and death are prevented (42). In the presence of the compatible solute glycine betaine—or its precursor choline—solutes are taken up by the action of different betaine-choline-carnitine transporters (BCCTs) (39). In the absence of compatible solutes in the medium, A. baumannii synthesizes the compatible solutes glutamate, mannitol, and trehalose *de novo* (36). Unfortunately, the molecular basis of sensing salinity and transmission of the signal to transcription and translation machineries is not understood.

CsrA is an mRNA binding protein that binds to the 5′ untranslated region (UTR) of mRNA as a symmetrical homodimer (43), thereby either blocking the ribosome binding site or inducing variations in mRNA stability (44–48) and thus causing global alterations in translation and regulation of the utilization of carbon sources (48, 49). The role of CsrA, however, is not restricted to carbon utilization, but CsrA also regulates translation on a global scale; this includes the regulation of physiological processes and stress responses involving regulation of key enzymes that confer resistance toward hyperosmotic stress (48, 50, 51). Recent studies revealed that deletion of the *csrA* gene from the genomes of the A. baumannii strains AB09-003 and 17961 resulted in an increase in osmotic tolerance (41). This study aimed to elucidate the basis for this observation by analyzing the ability to synthesize compatible solutes of strain AB09-003 and 17961 but also ATCC 19606, a model strain to study osmostress response in A. baumannii (36, 37, 40, 52, 53).

## RESULTS

### Utilization of carbon and energy sources in human urine by A. baumannii is dependent on CsrA.

**FIG 1:**
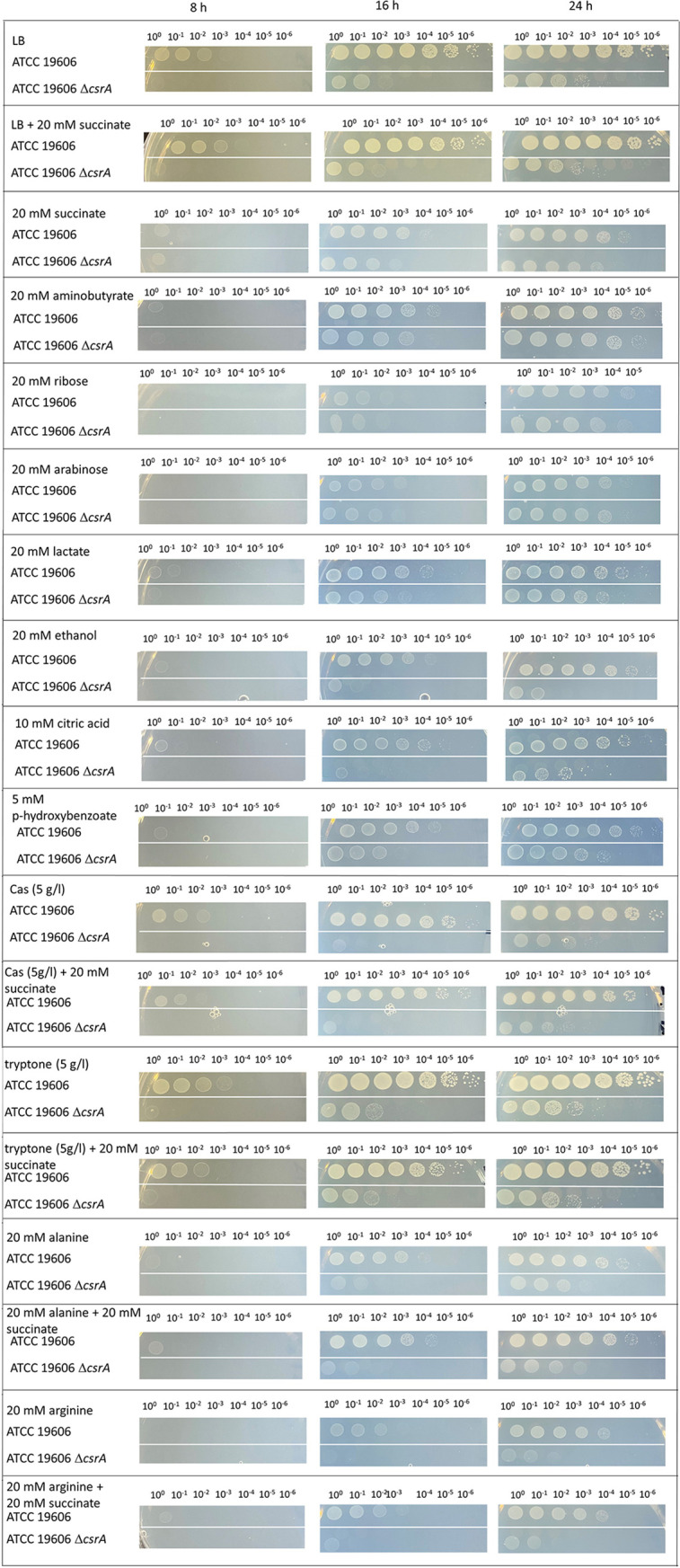
Growth of the A. baumannii ATCC 19606 Δ*csrA* mutant on solid medium with different carbon and energy sources. A. baumannii ATCC 19606 wild type cells and ATCC 19606 Δ*csrA* mutant cells were grown for 8 h in mineral medium with succinate as the carbon and energy source. Strains were centrifuged, washed twice with saline (0.9% NaCl), and adjusted with saline to an OD_600_ of 1. Serial dilutions from the cell suspensions (5 μl) were dropped onto the solid medium plates with the indicated carbon and energy sources. Growth was monitored after 8 h, 16 h, and 24 h of incubation at 37°C.

**FIG 2:**
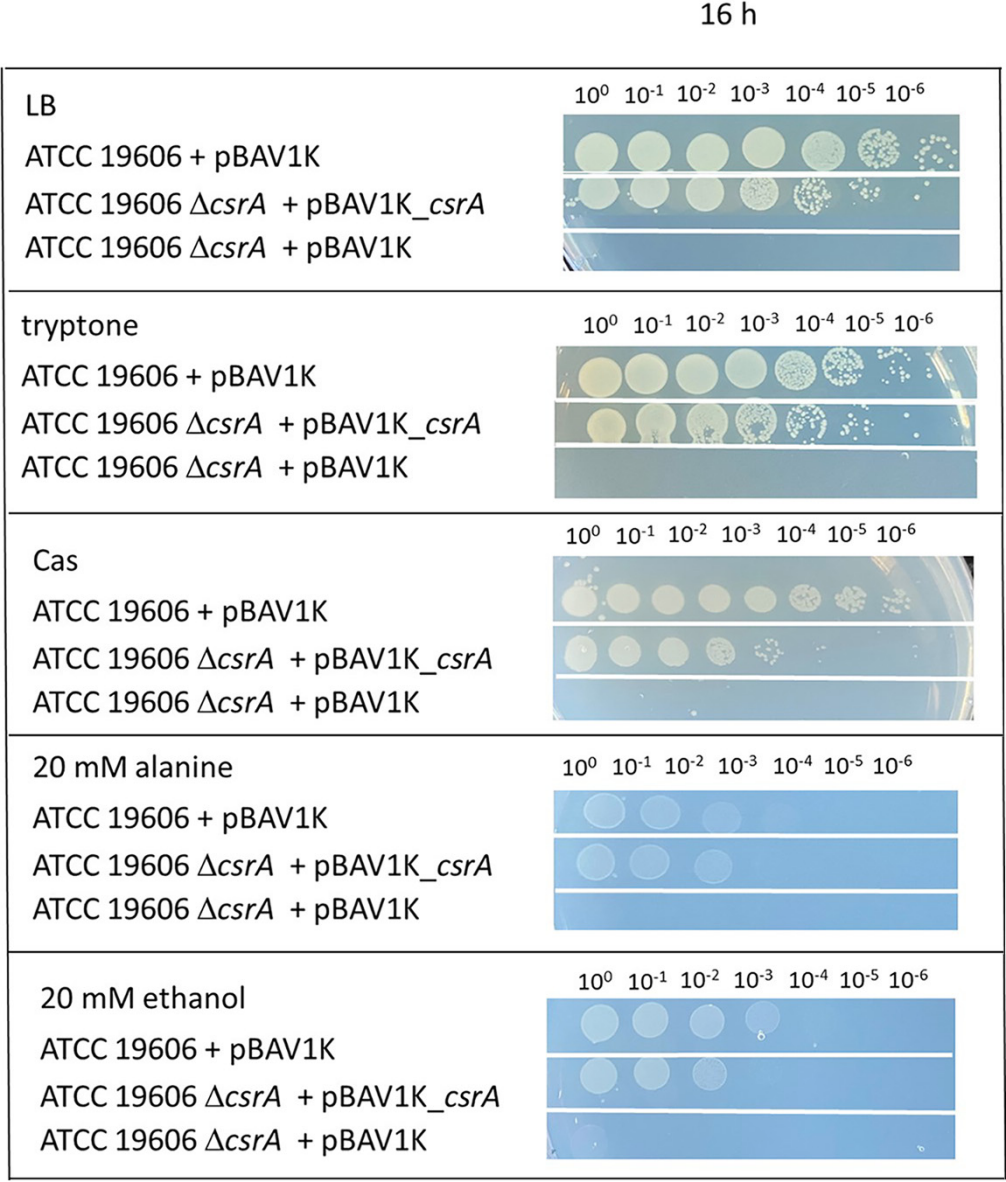
Effect of complementation of the Δ*csrA* mutant on growth in mineral medium with different carbon and energy sources. A. baumannii ATCC 19606 + pBAV1K, ATCC 19606 Δ*csrA* + pBAV1K, and Δ*csrA* + pBAV1K_*csrA* were grown for 8 h in mineral medium with succinate as the carbon and energy source and in the presence of 50 μg/ml kanamycin. Strains were centrifuged, washed twice with saline (0.9% NaCl), and adjusted with saline to an OD_600_ of 1. Serial dilutions from the cell suspensions (5 μl) were dropped onto solid medium (1.8%) in the presence of the indicated carbon and energy sources and in the presence of 50 μg/ml kanamycin. Growth was monitored after 16 h of incubation at 37°C.

To determine the role of CsrA in solute synthesis of A. baumannii, we generated a *csrA* deletion mutant of the type strain A. baumannii ATCC 19606 by an established insertion duplication mutagenesis protocol of Stahl et al. (14). This method is based on insertion of a *sacB*-*kan^R^* cassette in the *csrA* locus, followed by *csrA* segregation due to counterselection with sucrose. As reported earlier, a Δ*csrA* mutant of A. baumannii strains ATCC 17961 and AB09-003 (41), as well as *csrA* mutants of other bacteria (54), did not grow in complex media. A. baumannii strains 17961 and AB09-003 did not grow in LB or in tryptone or Müller-Hinton broth, but they used sugars such as arabinose and xylose and the organic acid acetate as carbon sources (41). Also, the growth of the ATCC 19606 Δ*csrA* mutant was impaired in LB medium (Fig. 1). We then tested for growth of the Δ*csrA* mutant on mineral medium plates with different carbon and energy sources (Fig. 1). Growth of the Δ*csrA* mutant and the wild type was comparable on succinate. The same was true for γ-aminobutyrate, which is fed into the TCC; growth on ribose, arabinose, and lactate was also not affected. In contrast, growth on ethanol, citric acid, and *p*-hydroxybenzoate, typical environmental carbon and energy sources, was impaired in the Δ*csrA* strain; the same was true for growth on Casamino Acids (CAS) and tryptone. A closer examination of the utilization of amino acids, potential carbon and energy sources for A. baumannii in urine, revealed that the Δ*csrA* mutant was impaired in growth with alanine and arginine. Interestingly, growth of the Δ*csrA* mutant on LB, tryptone, CAS, alanine, and arginine was not enhanced by the addition of succinate (Fig. 1). To exclude polar effects of the markerless mutation, the Δ*csrA* mutant was complemented with pBAV1K_*csrA*. Growth studies of the complemented Δ*csrA* mutant on LB, tryptone, CAS, alanine, and ethanol provided clear evidence that the impaired growth phenotypes of the Δ*csrA* mutant were complemented by providing the *csrA* gene in *trans* (Fig. 2).

**FIG 3:**
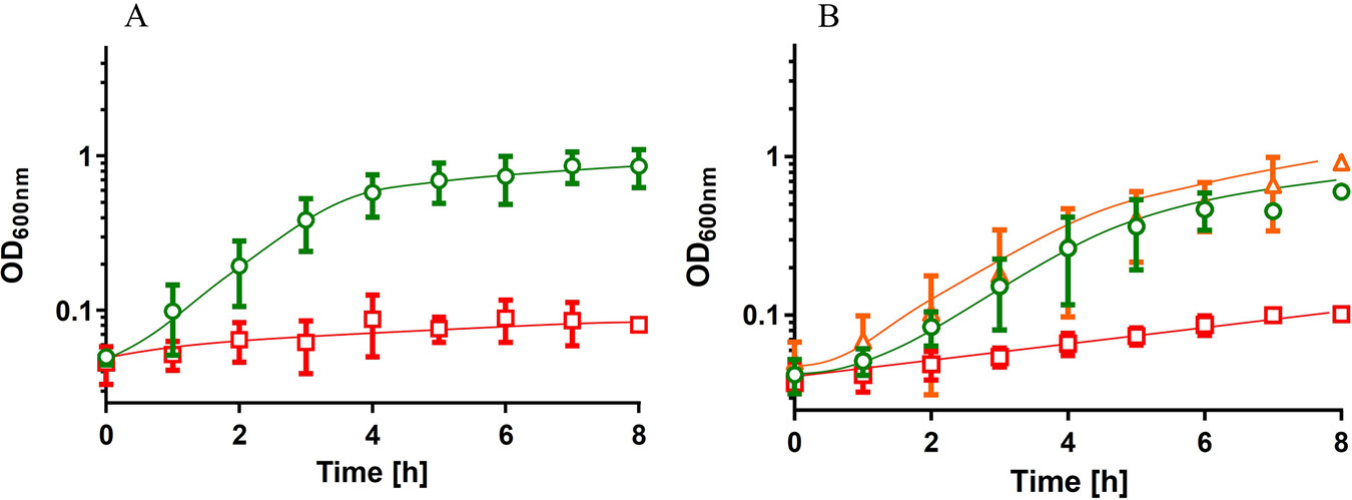
CsrA is essential for growth in human urine. Precultures were grown in mineral medium with succinate as the carbon source and washed in saline before inoculation of fresh prewarmed human urine to an initial OD_600_ of 0.05. (A) growth of A. baumannii ATCC 19606 (green circles) and Δ*csrA* (red squares) in human urine. (B) growth of the wild type ATCC 19606 + pBAV1K (green circles), the complemented strain Δ*csrA* + pBAV1K_*csrA* (orange triangles), and the Δ*csrA* mutant with the vector pBAV1K (red squares) in human urine in the presence of 50 μg/ml kanamycin. Error bars denote the standard deviation from the mean calculated from at least three biological replicates.

**FIG 4:**
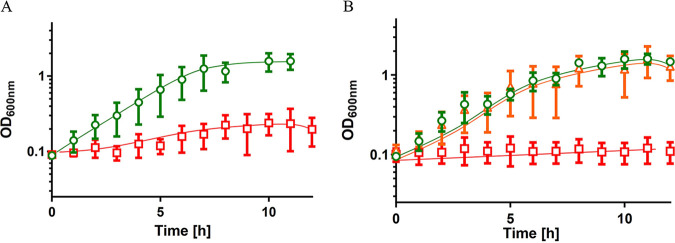
Deletion of *csrA* resulted in a thermo-sensitive phenotype. A. baumannii strains were grown overnight at 37°C in mineral medium with succinate. Strains harboring the pBAV1K plasmid were grown in the presence of 50 μg/ml kanamycin. The precultures grown in mineral medium with succinate were used to inoculate prewarmed medium to an initial OD_600_ of 0.1. (A) A. baumannii ATCC 19606 (green circles) and ATCC 19606 Δ*csrA* (red squares) were grown at 45°C. (B) ATCC 19606 + pBAV1K (green circles), ATCC 19606 Δ*csrA* + pBAV1K (red squares), and ATCC 19606 Δ*csrA* + pBAV1K_*csrA* (orange triangles) were grown at 45°C. Error bars denote the standard deviation of the mean, calculated from at least three biological replicates.

As can be seen in Fig. 3, the wild type grew in human urine. Growth started without a lag phase and proceeded with a growth rate of 0.5 h^−1^ until the final optical density of 0.73 ± 0.22 was reached after 6 h. The growth rate was slightly lower than the growth rate in mineral medium with CAS (0.9 h^−1^) or succinate (0.8 h^−1^). Interestingly, the Δ*csrA* mutant did not grow at all in human urine (Fig. 3A), but growth was restored by complementation with *csrA* in *trans* (Fig. 3B). Further phenotypic studies revealed a temperature-sensitive phenotype of the Δ*csrA* mutant during growth at high temperatures (45°C) (Fig. 4A). This growth impairment at high temperature was restored by complementation of the Δ*csrA* mutant (Fig. 4B). As reported earlier for A. baumannii strains 17961 and AB09-003, deletion of *csrA* led to reduced desiccation resistance and reduced virulence in a Galleria mellonella infection model (see Fig. S1 and S2 in supplemental material) (41).

### The strain-dependent hypersensitivity toward hyperosmotic stress is abolished by *csrA* deletion.

**FIG 5:**
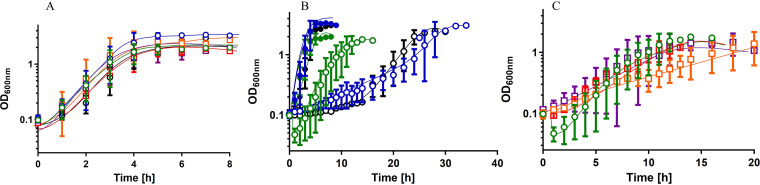
Growth of different A. baumannii strains in mineral medium in the presence and absence of 300 mM NaCl. (A) A. baumannii ATCC 19606 wild type (green circles) and Δ*csrA* (red squares), A. baumannii 17961 wild type (blue circles) and Δ*csrA* (orange squares), and AB09-003 wild type (black circles), and Δ*csrA* (purple squares) were grown overnight in mineral medium with succinate as the carbon and energy source. Precultures were used to inoculated prewarmed mineral medium with succinate to an initial OD_600_ of 0.1. (B) A. baumannii ATCC 19606 (green circles), A. baumannii 17961 (blue circles), and AB09-003 (black circles) were grown in mineral medium in the absence (solid symbols) or in the presence (open symbols) of 300 mM NaCl. (C) The growth of A. baumannii ATCC 19606 Δ*csrA* (red squares), A. baumannii 17961 Δ*csrA* (orange squares), and AB09-003 Δ*csrA* (purple squares) was compared to the growth of the A. baumannii ATCC 19606 type strain (green circles) in mineral medium with 300 mM NaCl. Error bars denote the standard deviation from the mean, calculated from at least three biological replicates.

Farrow et al. (41) reported earlier that deletion of *csrA* from the genome of strains 17961 and AB09-003 resulted in an enhanced growth upon osmotic upshift (200 mM NaCl) in the absence of compatible solutes in the extracellular medium (41), and it was suggested that CsrA of A. baumannii may be involved in (de)regulation of *de novo* synthesis of compatible solutes. We repeated the experiment with A. baumannii ATCC 19606 wild type cells and the Δ*csrA* mutant using the same medium (mineral medium with succinate and 200 mM NaCl) and also included the strains AB09-003 and 17961, kindly provided by Everett Pesci, ECU Brody School of Medicine at East Carolina University, USA. As observed before (41), Δ*csrA* mutants of strains AB09-003 and 17961 exhibited a reduced lag phase and an enhanced growth rate when exposed to 200 mM NaCl. In contrast, the Δ*csrA* mutant of strain ATCC 19606 exhibited a growth phenotype in high-salt medium comparable to the growth phenotype of wild type cells (Fig. S3). To elucidate the molecular basis for this strain-dependent difference, we compared the growth of the different strains and their ability to synthesize compatible solutes. For all further studies we used our standard medium (mineral medium with succinate [36]) and applied osmotic upshifts by the addition of 300 mM NaCl. No significant growth differences of the three wild type strains (ATCC 19606, 17961, and AB09-003) were observed during growth in mineral medium in the absence of high salt with succinate as the carbon source (Fig. 5A and B), and all strains reached their final optical density after approximately 4 h. The final optical density at 600 nm (OD_600_) of 17961 and AB09-003 cultures was higher (OD_600_ of ∼3) than the final optical density of a ATCC 19606 culture (OD_600_ of ∼2). However, the addition of 300 mM NaCl caused striking, strain-dependent differences. Osmotic upshift caused a prolonged lag phase (4 h) of ATCC 19606. The lag phase of strains 17961 and AB09-003 was even more extended (∼18 h) (Fig. 5B). Obviously, A. baumannii ATCC 19606 is capable of rapidly adapting to high osmotic upshift, whereas the other two strains are strongly impaired. Interestingly, deletion of *csrA* abolished the lag phase and led to immediate start of growth of the strains 17961 and AB09-003 (Fig. 5C).

### Strain-dependent differences upon hyperosmotic stress are abolished in the presence of glycine betaine.

**FIG 6:**
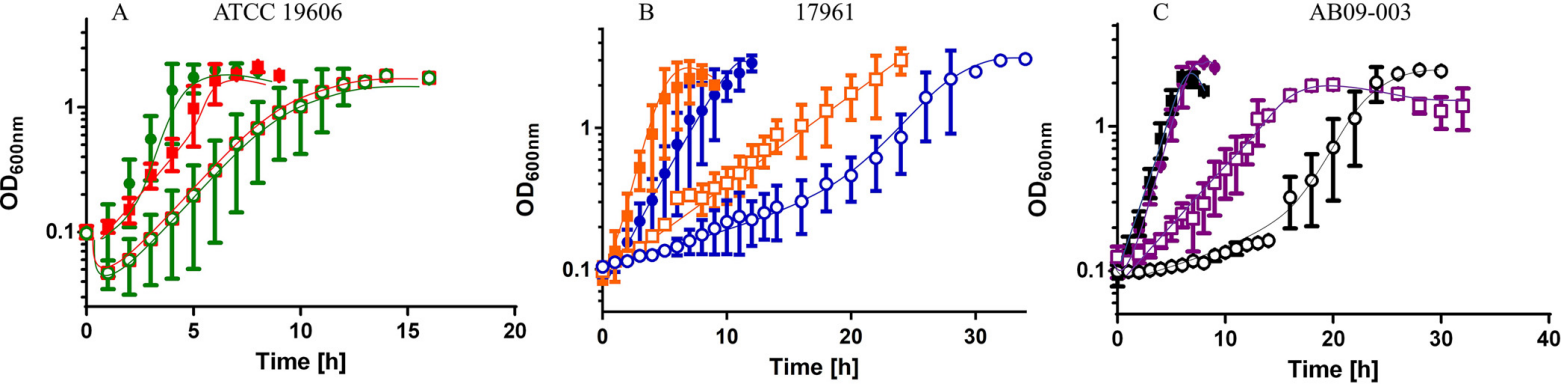
Growth of different A. baumannii strains and their corresponding Δ*csrA* mutants under hyperosmotic stress in the presence of the compatible solute glycine betaine. The A. baumannii wild type strain and Δ*csrA* strains were grown in mineral medium with 300 mM NaCl (open symbols) and with the addition of 1 mM glycine betaine (solid symbols). (A) growth of A. baumannii type strain 19606 (green circles) and Δ*csrA* (red squares) (B) A. baumannii type strain 17961 (blue circles) and Δ*csrA* (orange squares). (C) A. baumannii type strain AB09-003 (black circles) and Δ*csrA* (purple squares). Error bars denote the standard deviation from the mean, calculated from at least three biological replicates.

Under hyperosmotic stress, A. baumannii ATCC 19606 takes up glycine betaine as compatible solute, thereby repressing *de novo* synthesis of solutes (36). The lag phase of ATCC 19606 grown in mineral medium with 300 mM NaCl was reduced from approximately from 4 h to 2 h by the addition of glycine betaine (Fig. 6A), whereas the lag phase of the two other strains was reduced from 18 h to 2 to 4 h (Fig. 6B and C). Furthermore, no significant growth differences of the *csrA* deletion mutants and their corresponding wild type strains were observed. Taken together, the strain-dependent differences in growth with high salt are abolished by the addition of compatible solutes, suggesting that CsrA is involved in synthesis of compatible solutes.

### Synthesis of compatible solutes in A. baumannii AB09-003 and 17961 is repressed by CsrA.

**FIG 7:**
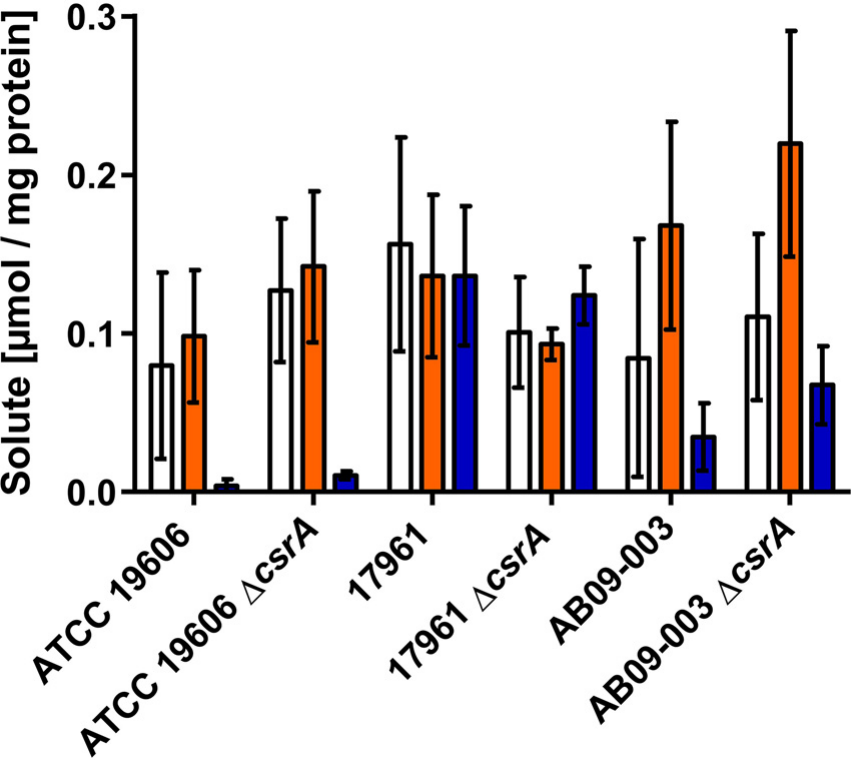
Solute pool of different A. baumannii strains and their corresponding Δ*csrA* mutants. All strains were grown in mineral medium with 300 mM NaCl until they reached an optical density of 0.9 to 1. Cells were harvested and solutes were extracted. The intracellular content of glutamate (white bar), mannitol (orange bar), and trehalose (blue bar) was quantified. Error bars denote the standard deviation, calculated from at least three biological replicates.

**FIG 8:**
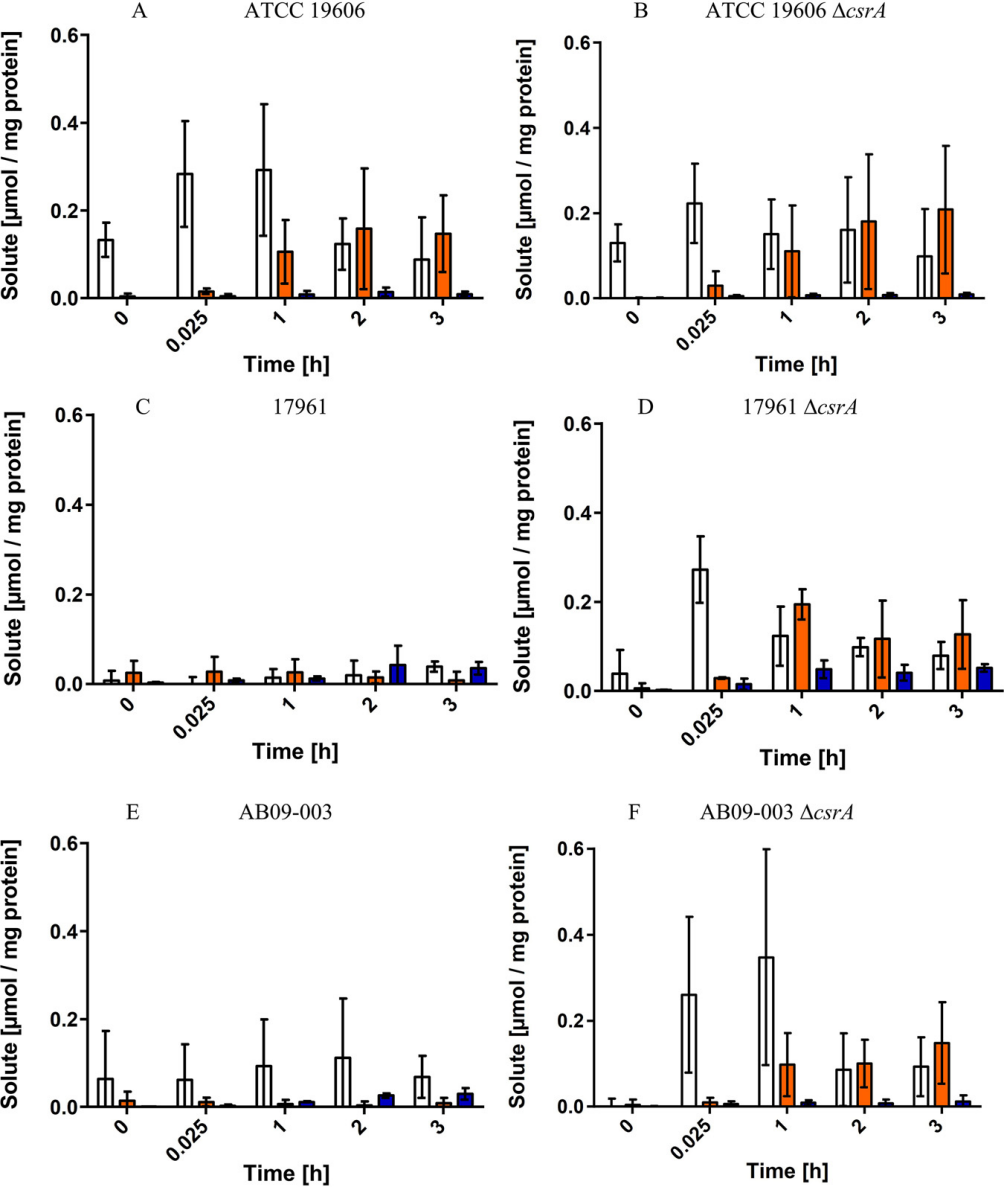
Solute synthesis of different A. baumannii strains and their corresponding Δ*csrA* mutants after osmotic upshift. A. baumannii ATCC 19606, 17961, and AB09-003 and their corresponding Δ*csrA* mutants were grown in mineral medium with succinate as the carbon and energy source. After they reached an optical density of 0.4 to 0.5, cells were exposed to 300 mM NaCl, indicated as time point = 0. Cells were harvested to the indicated time points, and the glutamate (white bars), mannitol (orange bars), and trehalose (blue bars) content was quantified. Error bars denote the standard deviation from the mean, calculated from at least three biological replicates.

To address a role of CsrA in solute synthesis, we analyzed the solute pool of the different strains and their corresponding Δ*csrA* mutants. The strains were grown in mineral medium with 300 mM NaCl and harvested in the exponential phase (OD_600_ of 0.9 to 1.1). Solutes were isolated and quantified (Fig. 7) as previously described (36). All three wild type strains synthesized glutamate, mannitol and trehalose but there were slight strain-dependent differences in the solute pool. While all strains accumulated glutamate in the range of 0.1 to 0.2 μmol/mg protein, the trehalose content of strains AB09-003 and 17961 was strongly increased (from 0.01 μmol/mg protein up to 0.05 to 0.13 μmol/mg) in comparison to strain ATCC 19606. This is especially interesting since trehalose is assumed to be essential for infection (25). Even though slight differences in the solute pool were observed, the overall osmo-stress response—accumulation of glutamate, mannitol and trehalose—was the same in all the strains. In contrast to our assumption, deletion of *csrA* did not result in significant differences of the solute pool in the strains (Fig. 7). Compatible solutes were synthesized in concentrations comparable to those of the wild type. However, it should be mentioned that cells were harvested in the exponential growth phase. We hypothesized that CsrA may repress the early steps in solute synthesis. To this end, we changed the experimental setup. The strains were grown to the early exponential phase (OD_600_ of 0.4 to 0.5), and osmotic upshift was induced by the addition of 300 mM NaCl. The solute pool was directly analyzed (Fig. 8). Just 15 min after the osmotic shock, A. baumannii ATCC 19606 synthesized glutamate, followed by mannitol and trehalose synthesis. The same holds true for the ATCC 19606 Δ*csrA* strain (Fig. 8A and B). In contrast, A. baumannii AB09-003 and 17961 did not accumulate glutamate or mannitol within the first hours after osmotic upshift, but deletion of *csrA* from these two strains led to glutamate synthesis directly after the osmotic upshift, followed by the synthesis of mannitol and trehalose. The differences in the glutamate content of strains 17961 and 17961 Δ*csrA* 15 min after osmotic upshift are significant (*P* = 0.0035), and the differences in mannitol content 1 h after osmotic upshift are significant as well (*P* = 0.0029). Unfortunately, this does not hold true for strain AB09-003 and the corresponding Δ*csrA* mutant, where the differences in glutamate content are not statistically significant due to rather high error bars. Nevertheless, the differences in the mannitol content between the wild type strain AB09-003 and the corresponding *csrA* mutant strain 2 h after osmotic upshift are significant (*P* = 0.04).Taken together, these findings lead to the conclusion that CsrA represses the synthesis of compatible solutes in the salt-sensitive strains AB09-003 and 17961.

## DISCUSSION

This work aimed to elucidate the role of the global posttranscriptional regulator CsrA of A. baumannii ATCC 19606 in cell physiology as well as in infection. As reported earlier, CsrA is essential for utilization of amino acids as a carbon source in A. baumannii (41). A similar observation was made in Escherichia coli, in which CsrA is essential for growth in medium with glycolytic carbon sources but not with pyruvate, which is decarboxylated and fed into the TCC (54). Our findings are in line with those of Farrow et al. (41), pointing out that CsrA of A. baumannii is critical for amino acid metabolism but not for metabolism of sugars. We additionally observed that growth of the Δ*csrA* mutant was also impaired on alcohols and aromatic compounds. The global regulatory effects of CsrA are crucial not only for carbon regulation and flow but also for infection. Studies with Vibrio cholerae revealed that CsrA is a positive regulator of the global virulence gene regulator ToxR (55), indicating that CsrA is also a global virulence regulator in V. cholerae. This is in line with the observation that CsrA is essential for pathogenesis of V. cholerae in a mouse model (55). We also observed reduced virulence of A. baumannii ATCC 19606 Δ*csrA*, in line with the data from strains 17961 and AB09-003 (41). Reduction of pathogenesis in a Δ*csrA* deletion mutant may also be due to interactions of CsrA with a global regulator, as observed in V. cholerae (55). However, adaptation to the human host requires global cell physiological changes, including the carbon metabolism. In particular, the amino acid metabolism pathways are reported to be required for A. baumannii AB5075 to grow in G. mellonella (25) and for persistence of A. baumannii ATCC 17978 in the mouse lung (27). This is consistent with the observation that histidine utilization promotes pathogenesis of A. baumannii ATCC 17978 in a murine model (56). The inability of the Δ*csrA* mutant to efficiently utilize amino acids may explain the reduced virulence in G. mellonella and the observed growth impairment in human urine, where amino acids are an abundant carbon sources (32). Supplementation of human urine with succinate did not restore growth of the mutant; the same was also true for supplementation of complex medium with succinate, indicating that growth impairment in complex media is not only due to the inability to grow with amino acids but is also due to deregulation of the central metabolic pathways—including the TCC (54).

In addition to the involvement in infection, CsrA represses the osmostress response in A. baumannii strains AB09-003 and 17961 (41). The hyperosmotic stress response can be divided into two main steps. The first step of the hyperosmotic stress response is the transient accumulation of potassium ions and synthesis of glutamate as a counterion; the second step is the exchange of potassium-glutamate against more suitable and nonpolar solutes such as glycine betaine, mannitol, or trehalose (36, 40, 42, 53, 57). Uptake of glycine betaine is energetically favored over *de novo* synthesis of mannitol and trehalose (36). It is assumed that potassium glutamate acts as the second messenger, which triggers uptake or synthesis of compatible solutes in bacteria (58). This observation seems to be true for A. baumannii strain ATCC 19606, where glutamate, mannitol, and trehalose are synthesized in a strict temporal order after an osmotic upshift (40). However, in the A. baumannii strains AB09-003 and 17961, CsrA represses the ability to synthesize the compatible solutes glutamate and mannitol within the first hours after osmotic upshift. This explains the increased sensitivity of these two strains against hyperosmotic stress, but the interaction partner of CsrA remains unknown. Impairment of the synthesis of all compatible solutes is most likely explained by deregulation of an early step of the osmostress reaction cascade, such as the uptake of potassium ions or glutamate synthesis. We favor the idea of a CsrA-mediated inhibition of glutamate synthesis over a deregulation of potassium uptake. This seems to be more likely since CsrA is the major regulator of amino acid metabolism. Moreover, this hypothesis is further supported by the observation that CsrA is not involved in the uptake of glycine betaine under hyperosmotic stress, which is usually activated via interactions of the transporter with potassium ions (59).

## MATERIALS AND METHODS

### Bacterial strains and culture conditions.

E. coli DHα was used for the generation of recombinant plasmids and was grown in complex medium (LB) (60). For generation of a *csrA* deletion, A. baumannii ATCC 19606 was grown in complex medium. For all further studies, the three A. baumannii strains and their corresponding *csrA* deletion mutants were grown in phosphate-buffered mineral medium with succinate as the carbon source, as described before (36). For growth studies on solid medium, A. baumannii precultures were grown for 8 h in mineral medium with succinate as the carbon and energy source. The cells were harvested by centrifugation, washed twice in saline (0.9% NaCl), and adjusted to an OD_600_ of 1. Serial dilutions of the cell suspensions were performed with saline, and 5 μl of the dilutions were dropped onto solid medium. Growth in liquid medium was performed at 37°C and 130 rpm. If required, 50 μg/ml (A. baumannii) or 20 μg/ml (E. coli) kanamycin was added for selection. Growth experiments in mineral medium were started by inoculation of prewarmed medium (100 ml) with an overnight culture to an OD_600_ of 0.1. Growth was followed by measuring the OD_600_.

### Markerless mutagenesis.

To determine the role of CsrA in solute synthesis of A. baumannii, we generated a *csrA* deletion mutant of the type strain A. baumannii ATCC 19606. Markerless deletion of *csrA* (HMPREF0010_03075) was performed using the established insertion-duplication mutagenesis system described before (14) with slight modifications. Briefly, 1,500 bp up- and downstream of the *csrA* locus were amplified via PCR using the primer pair *csrA*_upstream forward (fw) and reverse (rev) and *csrA*_downstream fw and rev (primers used are listed in Table S1). The vector pBIISK_*sacB*_*kanR* was amplified using the forward primer and the reverse primer (Table S1). The resulting PCR fragments were joined using Gibson assembly (Gibson assembly master mix; New England Biolabs, Ipswich, MA, USA), resulting in the recombinant plasmid PBIISK_*sacB*_*kanR*_Δ*csrA*. A. baumannii ATCC 19606 was transformed with the plasmid via electroporation (2.5 kV, 200 Ω, and 25 μF). Transformants were selected on complex medium (LB, 1.8% agar) in the presence of 50 μg/ml kanamycin. Negative selection was applied by the addition of 20% sucrose in mineral medium with 20 mM succinate as the carbon source. Deletion of *csrA* was confirmed by PCR and sequencing.

### Complementation.

For genetic complementation of the ATCC 19606 Δ*csrA* mutant, the *csrA* gene, and the promoter region upstream of *csrA* were amplified from genomic DNA of A. baumannii 19606 via PCR using the primers *csrA*_up fw and rev. The vector pBAV1K was amplified with primers pBAV1K fw and rev (Table S1). The resulting PCR products were joined using Gibson assembly, resulting in the plasmid pBAVK1K_*csrA*. A. baumannii ATCC 19606 was transformed with the vector pBAV1K, and A. baumannii Δ*csrA* was transformed either with the vector pBAV1K or the plasmid pBAVK1K_*csrA* via electroporation (2.5 kV, 200 Ω, and 25 μF). Transformants were selected on mineral medium plates with succinate as the carbon source in the presence of 50 μg/ml kanamycin.

### Preparation of urine and growth.

Human urine was collected from 10 volunteers (male and female) with an age of 20 to 30. The urine was pooled, centrifuged (10 min, 8,000 rpm, 4°C), and sterilized via filtration (0.45-μm filter). For growth experiments, A. baumannii strains were grown in 5 ml mineral medium with succinate overnight. The overnight cultures were centrifuged (4,700 rpm, 5 min) washed, and resuspended in sterile saline. The cell suspensions were used to inoculate prewarmed urine (50 ml) to an initial OD_600_ of 0.05. Cells were grown at 37°C and 130 rpm, and growth was monitored photometrically by measuring the optical density at 600 nm. For supplementation studies, the urine was supplemented with 20 mM succinate.

### Isolation and characterization of the compatible solute pool.

For isolation of compatible solutes, cells were grown in mineral medium with 300 mM NaCl until they reached an OD_600_ of 0.9. Additionally, solutes were determined directly after osmotic upshift. Therefore, cells were grown in mineral medium to the exponential growth phase (OD_600_ of 0.4 to 0.5), and hyperosmotic stress was applied by the addition of 300 mM NaCl. Cells were incubated (37°C, 130 rpm) and partly harvested over a time period of 3 h. Solute extraction was performed with chloroform and methanol as described before (36). Mannitol, trehalose, and glutamate were determined enzymatically (trehalose assay kit K‐TREH, l‐glutamic acid assay kit K‐GLUT, and d-mannitol assay kit from Megazyme, Bray, Ireland) (36).

### Statistical analysis.

The standard deviation from the mean was calculated from at least three biological replicates. Differences in the compatible solute pool were analyzed using an unpaired *t* test (GraphPad Prism 6 Software). *P* values of ≤0.05 were considered statistically significant.
