# Supplementary material for: CsrA Coordinates Compatible Solute Synthesis in Acinetobacter baumannii and Facilitates Growth in Human Urine
Source: Microbiol Spectr. 2021 Nov 3;9(3):e01296-21. doi: 10.1128/Spectrum.01296-21 (PMC8567240; doi:10.1128/Spectrum.01296-21)
Supplement: SUPPLEMENTAL FILE 1 — Supplemental material. Download SPECTRUM01296-21_Supp_1_seq12.pdf, PDF file, 0.4 MB [file spectrum01296-21_supp_1_seq12.pdf]

SUPPLEMENTAL MATERIAL

CsrA coordinates compatible solute synthesis in *Acinetobacter baumannii* and facilitates growth in human urine

Josephine Joy Hubloher, Kim Schabacker, Volker Müller, Beate Averhoff*

Department of Molecular Microbiology & Bioenergetics, Institute of Molecular Biosciences, Goethe-University Frankfurt am Main, Germany;

Running title: CsrA in *A. baumannii* ATCC 19606

*Correspondence to: Beate Averhoff, Department of Molecular Microbiology & Bioenergetics, Institute of Molecular Biosciences, Goethe-University Frankfurt am Main, Max-von-Laue-Str. 9, 60438 Frankfurt, Germany. Tel.: + 49 69 79829509. E-mail address: averhoff@bio.uni-frankfurt.de

SUPPLEMENTAL MATERIAL

Table S1 Primers used in this study.

*csrA*_upstream fw	CCCGGGGGATCCACTAGTTCCGGTACTTCTATGGGTAC
*csrA*_upstream rev	CAACTTCATAGTTTATCTCCTTGCTAAACG
*crsA*_downstream fw	GGAGATAAACTATGAAGTTGTTCTCTCCC
*crsA*_downstream rev	CCGCGGTGGCGGCCGCTCTAGATATTCTTATGTAGTGTAATGAC
pBIISK_*sacB*_*kan*^{R} fw	TAGAGCGGCCGCCACCGC
pBIISK_*sacB*_*kan*^{R} rev	GAACTAGTGGATCCCCCGGGC
*csrA*_up fw	AGAATTTGACGTCGAATTCGCACGTGAAGTTTCAACAC
*csrA*_up rev	CCTGAGGCCTGCAGCGGCCGCTAACGATTGAAGTTTTTCTG
pBAV1k fw	CGGCCGCTGCAGGCCTCA
pBAV1k rev	CGAATTCGACGTCAAATTCTATCATAATTGTGGTTTCAAAATCGGCTC

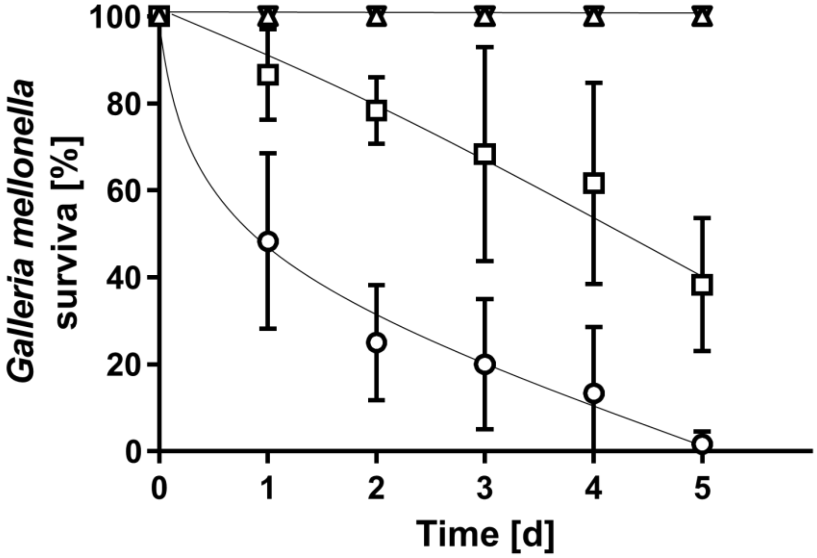

Fig. S1 *Galleria mellonella* killing. *A. baumannii* ATCC 19606 (○) and Δ*csrA* (□) were grown in mineral medium with succinate (20 mM) to an OD_{600nm} = 0.5. Bacteria were washed twice with saline (0.9 % NaCl) and 10 µl of the cell suspension (with approximately $5*10^6$ bacteria) where injected into the last proleg of preselected *G. mellonella* caterpillars (weight range between 0.35-0.45 g). The control groups were injected with or 10 µl saline (△) or were not injected at all (▽). Caterpillars were incubated in the dark over 5 day at 37 °C and the number of survived animals was determined. Caterpillars were considered dead if they did not respond towards gentle poking. Error bars denote the standard deviation calculated from at least three biological replicates.

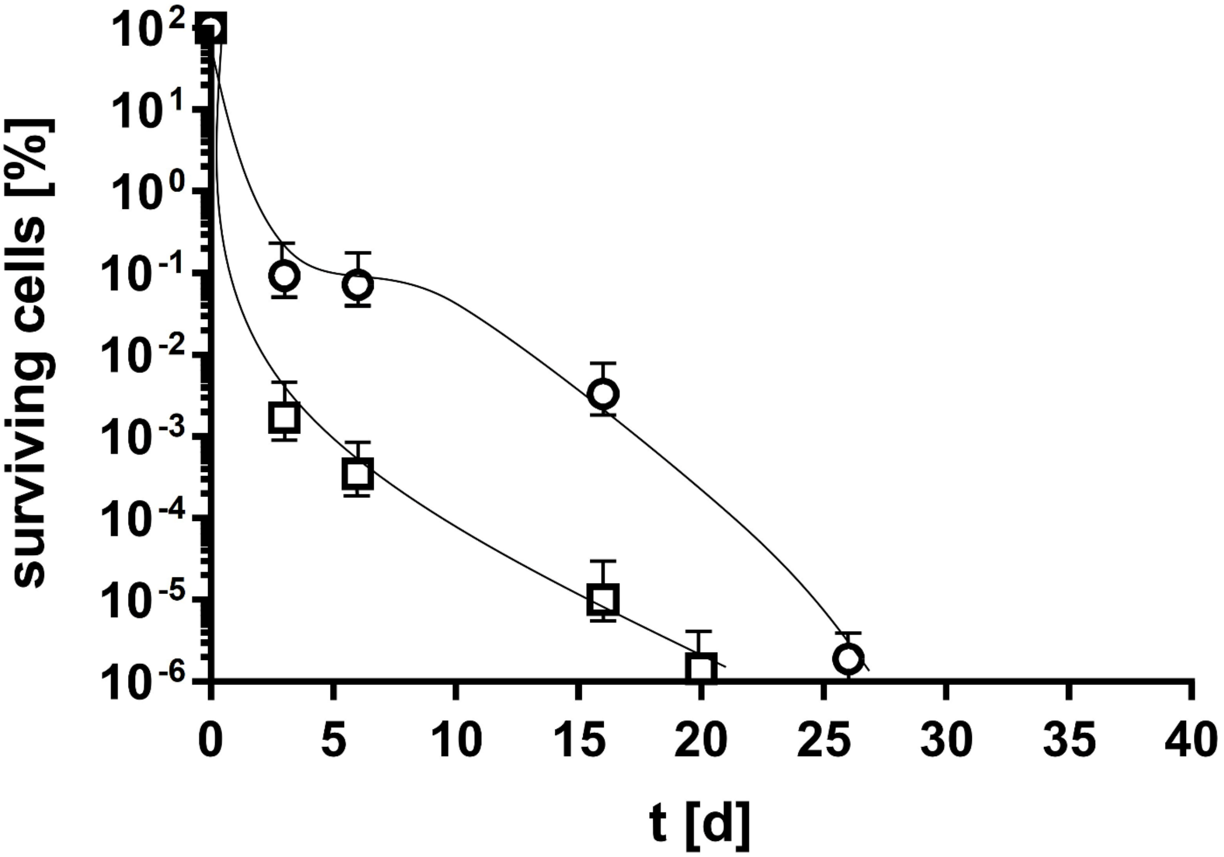

Fig. S2 Desiccation resistance of *A. baumannii* ATCC 19606 and Δ*csrA*. An overnight culture of *A. baumannii* ATCC 19606 (O) and Δ*csrA* (☐)were grown in mineral medium with 20 mM succinate. 1 ml of the overnight culture was harvested and washed twice in saline (0.9 % NaCl). Cells were adjusted to an OD_{600nm} = 2 and 20 µl of the cell suspension was spotted on small polycarbonate filters (Nuclepore Track-Etch Membrane, 13 mm, 0.4 µm). The cell suspension was dried in a climate chamber (31% relative humidity and 22°C). Bacterial survival was monitored *via* recovery of the cells from the filters and afterwards plating the cells on mineral medium agar for determination of number of colony forming units. Error bars denote the standard deviation calculated from at least three biological replicates.

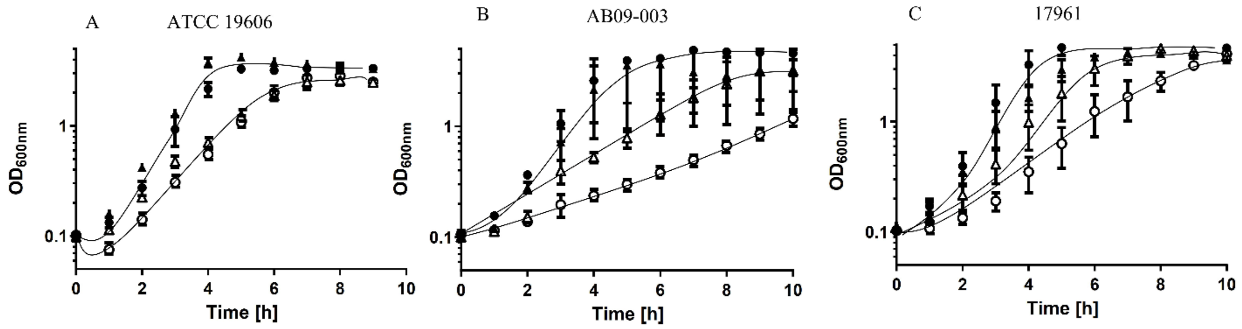

Fig. S3 Growth of *A. baumannii* ATCC 19606 and Δ*csrA* mutant in mineral medium with 200 mM NaCl according to Farrow *et al.*. *A. baumannii* ATCC 19606 (A), AB09-003 (B) and 17961 (C) wildtype strain (circle) and the Δ*csrA* strains (squares) were grown overnight in mineral medium with succinate as carbon source. Overnight cultures were used to inoculated prewarmed mineral medium with succinate in absence (closed symbols) or presence of 200 mM NaCl (open symbols). Error bars denote the standard deviation calculated from at least three biological replicates.
